# Prevalence and Impact of Cytomegalovirus Primary Infection and Reactivation on Graft Function in Post-Renal Transplant Recipients

**DOI:** 10.7759/cureus.74483

**Published:** 2024-11-26

**Authors:** Saravana Priya J K, Padma Kumari J, Secunda Rupert, Ramani C P

**Affiliations:** 1 Microbiology, Madras Medical College, Rajiv Gandhi Government General Hospital, Chennai, IND; 2 Regenerative Medicine and Research, Stanley Medical College, Chennai, IND

**Keywords:** acute and chronic dysfunction, acute cellular rejection, antibody-mediated rejection, cmv, cmv igg avidity, graft function, post-renal transplant recipients, primary infection, reactivation

## Abstract

Introduction

Cytomegalovirus (CMV) is often associated with mortality and significant morbidity following renal transplantation leading to graft rejection or dysfunction. Primary CMV infection refers to the first detection of the virus in a person who has no prior evidence of CMV exposure before transplantation. CMV has a unique property called latency. After the initial infection, CMV can enter a dormant state within the body, residing in myeloid cells without causing active disease. CMV reactivation is likely when a latent CMV infection switches to a lytic phase of replication, which can be detected using IgG avidity ELISA.

Aims and objectives

This study aims to assess the prevalence of primary CMV infection and reactivation in renal transplant recipients, evaluate the impact of CMV infection on graft function following transplantation, and identify the risk factors and comorbidities associated with CMV-related graft rejection.

Methodology

During the study period from March 2020 to November 2021, blood samples were collected from 46 CMV-positive (by PCR) renal transplant recipients, and serum was separated and stored. IgG avidity ELISA test was performed, which served as a valuable tool to differentiate primary infection from reactivation due to difference in binding strength where low binding strength (low avidity<30%) indicated primary infection and high binding strength (high avidity>40%) indicated reactivation. All these patients were followed up to study the impact of CMV on graft functions.

Results

The age-wise distribution of patients shows a maximum number of cases under 40 years. The gender distribution of cases shows a higher preponderance of males (76%) compared to females (24%). The clinical presentation showed CMV syndrome as the most common (50%), followed by CMV colitis (37%), CMV nephritis (9%), CMV pneumonitis, CMV esophagitis, and CMV duodenitis, each comprising 2%. After performing the IgG avidity test, CMV infection with maximum cases of reactivation (87%) followed by primary infection (13%) was observed. The investigations related to renal dysfunction such as serum creatinine showed >3 mg/dL (85% of cases), 2.1-3 mg/dL (4.33% of cases), 1.6-2 mg/dL (2% of cases), 1-1.5 mg/dL (4.33% of cases) in decreasing order. Normal urea values are seen in 9% of cases followed by the range between 24 and 55 mg/dL in 67% and >100% in 24% of cases. The graft rejection based on the biopsy report showed that acute cellular rejection (ACR) (72%) was higher followed by antibody-mediated rejection (ABMR) with 15% and then ACR + ABMR with 4%. No rejection was found in 9% of cases. Renal dysfunction showed a higher preponderance to chronic graft dysfunction (67%) followed by acute graft dysfunction (24%) and stable graft function among 9% of cases. A comparison of graft dysfunction in primary infection/reactivation was assessed, and it was found that acute graft dysfunction was more common in primary infection. In the case of reactivation, chronic graft dysfunction was more common.

Conclusion

This study focuses on the microbiological dimensions and the critical role of CMV antibody screening. It underscores the necessity of vigilant monitoring and prophylactic antiviral therapy to reduce CMV infection risks and enhance patient outcomes. It also highlights the use of IgG avidity testing to differentiate between primary infection and reactivation, facilitating timely and effective interventions to prevent graft dysfunction and rejection.

## Introduction

Cytomegalovirus (CMV) is a genus of viruses in the order Herpes virales, in the family Herpes viridae, in the sub-family Beta Herpes virinae, and is responsible for causing diseases in humans. Severe CMV infections are frequently found in immunosuppressed adults. The prevalence of CMV was around 12.4% in post-renal transplant recipients [1]. Primary infections suppress both cell-mediated and humoral immunity, causing the disease to be significantly more severe in immunosuppressed individuals compared to those with intact immune systems [2].

These infections are classified as early (occurring within 100 days after transplantation) and late (occurring after 100 days following transplantation) [3]. CMV infection is of major concern in the immune competent as well as in categories of immune-compromised individuals such as neonates, pregnant women, recipients of bone marrow and other organ transplants, and individuals having immunodeficiency disorders [4].

Primary CMV infection is defined as the detection of CMV infection in an individual for the first time who has no evidence of CMV exposure before transplantation. Latent infection happens after the initial immune response where the virus persists in a latent state in myeloid lineage cells and employs various mechanisms to evade the immune system and survive. CMV reactivation is likely when a latent CMV infection switches to a lytic phase of replication and if the two viral strains (prior and current strain) are found to be indistinguishable either by using a variety of molecular techniques or by sequencing specific regions of the viral genome that examine genes known to be polymorphic. Reinfection is defined as the detection of a CMV strain that is distinct from the strain that caused the initial infection. Recurrent infection is defined as a new CMV infection in a patient with previous evidence of CMV infection, where the virus has not been detected for at least 4 weeks during active surveillance. Recurrent infection may result from reactivation of latent virus (endogenous) or reinfection (exogenous). Secondary infection occurs when the patient acquires CMV infection for a second time, which may be due to reinfection, reactivation, or a recurrent infection. Active infection is defined by the presence of viral replication, diagnosed by growing the virus in vitro; by the discovery of intracytoplasmic and intranuclear inclusions, which are characteristics of the virus; by viral identification via tissue staining of biopsy material; or by the discovery of evidence of viral replication detected by antigenemia assay or molecular methods [5,6].

CMV produces complications that include pneumonia, colitis, retinitis, hepatitis, and CMV-related rejection of allograft, and sometimes the patient also presents with prolonged fever (CMV syndrome) [7]. Hence, this study provides insights into the prevalence of primary CMV infection/reactivation and its impact on graft function, along with the identification of other risk factors and comorbidities, which are essential for improving patient outcomes and optimizing treatment protocols.

## Materials and methods

This prospective observational study includes 46 CMV PCR-positive post-renal transplant recipients in whom the impact of CMV on graft functions and the ability to differentiate primary from reactivated CMV infections were analyzed by using anti-CMV IgG antibodies avidity enzyme immunoassay. Other risk factors and co-morbidities associated with graft rejections were also evaluated for one year and eight months at the Institute of Microbiology after obtaining approval from the Institutional Ethics Committee. Informed consent was obtained from post-renal transplant recipients affected by CMV infection. Patients above 18 years of age and CMV PCR-positive post-renal transplant recipients were included in the study. PCR was done in the Department of Regenerative Medicine and Research, Stanley Medical College for all patients showing clinical presentation suggestive of CMV infection.

The test procedure involves the collection of 5-7 mL of blood samples in a red vacuum container following standard precautions from post-renal transplant recipients. The blood sample was centrifuged at 5000 rpm for five minutes at room temperature, and the serum was separated. Stored the serum sample at -70°C. After the collection of all the samples, IgG avidity testing (enzyme immunoassay for the detection of anti-CMV IgG antibody avidity in human serum, ENZYWELL Cytomegalovirus IgG REF 91010/AVI/IMM (96 tests) - manufactured by DIESSE Diagnostica Senese, Via delle Rose, 53035 Monteriggioni (Siena), Italy) was performed according to the manufacturer’s instructions to differentiate between primary infection (low avidity) and reactivation (high avidity). All these patients were followed up for their graft function status, and graft dysfunction was assessed through renal biopsy.

Data processing and statistical analysis of the patient risk factors were done using IBM SPSS Statistics for Windows, Version 26 (Released 2019; IBM Corp., Armonk, New York, United States) [8]. Frequency and percentage analysis were used for categorical variables; mean and standard deviation were used for continuous variables. With a 95% confidence interval, a p-value of less than 0.05 was considered statistically significant.

## Results

Clinical presentation among the study population (N=46)

**Figure 1:**
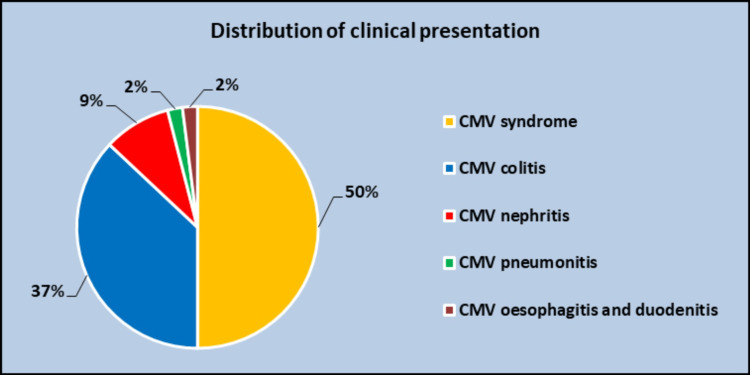
Distribution of clinical presentation (P<0.001)

The comparison of various clinical presentations in the study population is shown in Figure 1.

Co-morbidities among the cases (N=46)

**Figure 2:**
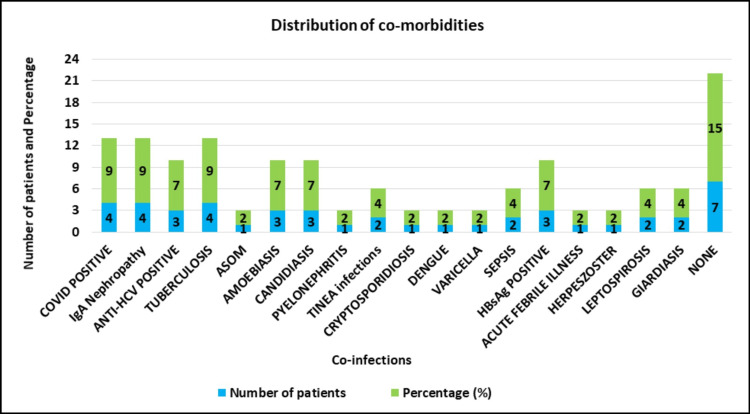
Distribution of co-morbidities among the cases (N=46, P=0.014)

Numerous co-morbidities among the study population were analyzed, which showed COVID-19, IgA nephropathy, and tuberculosis as the most common co-existing illness along with CMV infection (Figure 2).

Types of transplantation among the cases (N=46)

Out of 46 post-renal transplant recipients, most received live donor transplants compared to cadaveric transplants (Table 1).

**Table 1:** Types of transplantation among the cases N represents the exact number (frequency) and the percentage is expressed in a bracket.

Type	N (%)
Live donor	41 (89)
Cadaveric donor	5 (11)

Investigations done in post-renal transplant recipients among the cases (N=46)

The following investigations were compared in 46 post-renal transplant recipients, which showed that 31 (67%) had urea levels in the range of 24-99, and 39 (85%) had serum creatinine levels of more than 3. Among 46 recipients, 42 (91%) showed leukocytosis while 4 (9%) showed normal leukocyte count (Table 2). 

**Table 2:** Investigations done in post-renal transplant recipients among the cases (N=46) N represents the exact number (frequency) and the percentage is expressed in a bracket.

Investigations	N (%)
Urea	
6-24 mg/dL	4 (9)
24-99 mg/dL	31 (67)
More than 100 mg/dL	11 (24)
Creatinine	
Less than 1 mg/dL	0
1-1.5 mg/dL	2 (4.33)
1.6-2 mg/dL	1 (2)
2.1-2.5 mg/dL	2 (4.33)
2.6-3 mg/dL	2 (4.33)
More than 3 mg/dL	39 (85)
Total leukocyte count	
Normal count (4000-10,000)	4 (9)
Leukocytosis (>10,000)	42 (91)

CMV infection based on IgG avidity index ELISA (primary infection/reactivation) in the cases (N=46)

Reactivation (87%) was prevalent among 46 post-renal transplant recipients (Table 3).

**Table 3:** CMV infection based on IgG avidity index ELISA (primary infection​​​​​​​/reactivation) in the cases (N=46) N represents the exact number (frequency) and the percentage is expressed in a bracket () *Medium degree of avidity (borderline) and the test can be repeated after a few weeks

IgG Avidity index	N (%)
<30% (Primary)	6 (13)
30-40% (Borderline)*	0
>40% (Reactivation)	40 (87)

Graft rejection among the cases based on biopsy report (N=46)

Immune responses in the recipients were analyzed, and the report is shown in Table 4.

**Table 4:** Graft rejection among the cases based on biopsy report N represents the exact number (frequency) and the percentage is expressed in a bracket. ACR, acute cellular rejection; ABMR, antibody-mediated rejection

Biopsy report	N (%)
ACR	33 (72)
ACR + ABMR	2 (4)
ABMR	7 (15)
No rejection	4 (9)

Renal dysfunction among the cases (N=46)

Among 46 post-renal transplant recipients, chronic graft dysfunction was predominantly seen followed by acute graft dysfunction (Table 5).

**Table 5:** Renal dysfunction among the cases N represents the exact number (frequency) and the percentage is expressed in a bracket ()

Renal dysfunction	N (%)
Acute graft dysfunction	11 (24)
Chronic graft dysfunction	31 (67)
Stable graft function	4 (9)

Comparison of graft dysfunction in primary CMV infection and reactivation in post-renal transplant recipients (N=46)

**Figure 3:**
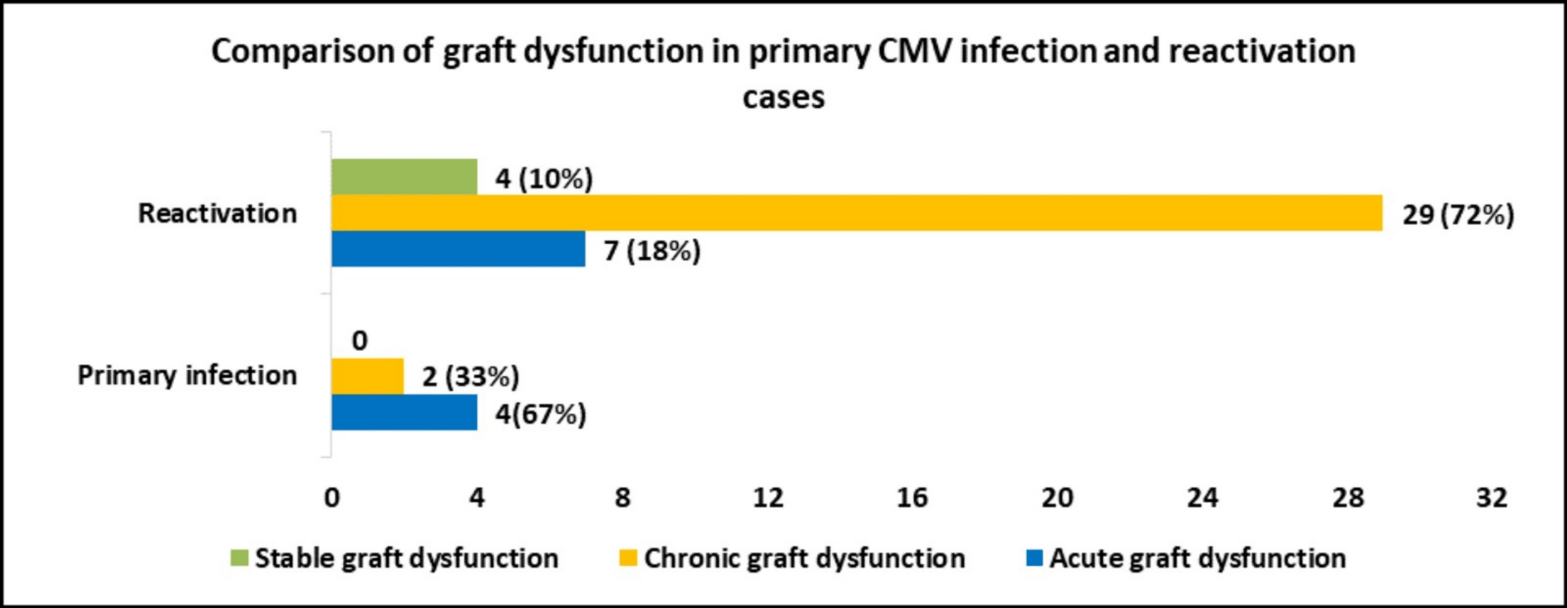
Comparison of graft dysfunction in primary CMV infection and reactivation in post-renal transplant recipients (P<0.001)

Graft dysfunctions were compared with primary CMV infection and reactivation, which showed that acute graft dysfunction was more common in primary infection, and chronic graft dysfunction was more common in reactivation (Figure 3).

Comparison of ACR and ABMR with primary CMV infection and reactivation in post-renal transplant recipients (N=46)

**Figure 4:**
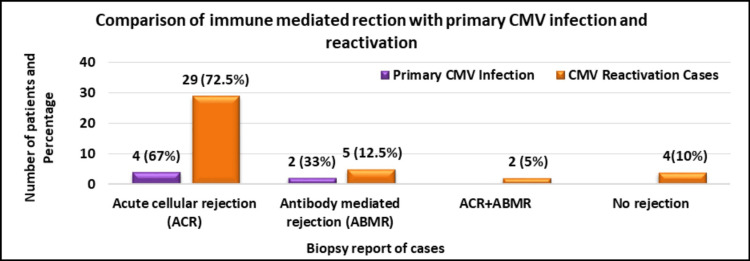
Comparison of ACR and ABMR with primary CMV infection and reactivation (P<0.001) ACR, acute cellular rejection; ABMR, antibody-mediated rejection

The immune response in recipients was compared with primary CMV infection and reactivation, which showed that acute cellular rejection (ACR) was more common in both primary infection and reactivation (Figure 4).

Comparison of immune-mediated rejection with acute, chronic dysfunction and stable graft function among the cases (N=46)

**Figure 5:**
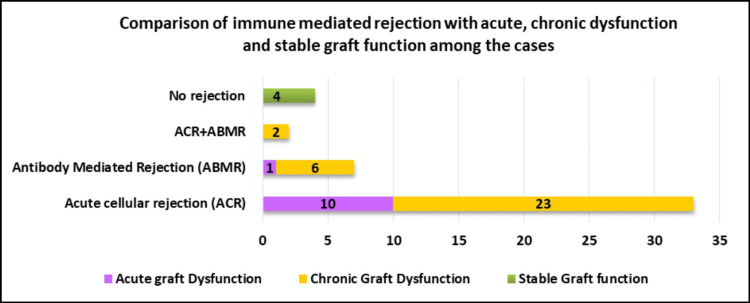
Distribution of immune-mediated rejection in acute, chronic graft dysfunction and stable graft function (P<0.001)

Figure 5 shows that ACR was more common in both acute and chronic graft dysfunction.

## Discussion

The present study analyzed CMV infections among the CMV PCR-positive post-renal transplant recipients using anti-CMV IgG antibodies avidity enzyme immunoassay to differentiate primary CMV infections from reactivation.

Out of 46 post-renal transplant recipients, the majority of patients (33, 72%) fell under the age group of <40 years, 12 (26%) patients were between 40 and 60 years, and one (2%) patient was above 60 years of age [9]. Analyzing the gender predominance, 35 (76%) of them were males and 11 (24%) of them were females [10].

Evaluating the clinical presentations of CMV infection, most of them (50%) manifested as CMV syndrome, followed by (37%) CMV colitis, and (9%) CMV nephritis [11,12].

Assessing the comorbidities among the recipients, COVID-19 positivity, IgA nephropathy, and tuberculosis were predominantly identified in 4 (9%) patients each, followed by Anti-HCV positivity, HBsAg positivity, amoebiasis, and candidiasis, which were found in 3 (7%) patients each [13].

Living donor transplant recipients had a decreased risk of graft failure than cadaveric donors [14]. The majority (42, 91%) showed leukocytosis while four (9%) showed normal leukocyte count [15].

In our study, among 46 post-renal transplant recipients, a maximum of 39 patients (85%) showed serum creatinine levels of more than three [16]. Among renal transplant recipients in our study, 31 (67%) patients showed urea levels in the range of 24-99. Blood urea nitrogen (BUN) and serum creatinine are markers that are typically measured repeatedly over time for patients who have undergone renal transplantation. These markers evaluate how well the kidneys are functioning following renal transplantation [17].

IgG avidity ELISA revealed that 40 (87%) patients had an avidity index of more than 40%, which is due to immunosuppressive agents following solid organ transplantation (SOT), which disrupted the immune function and caused viral reactivation, particularly in the first six months after transplant [18].

An analysis of the prevalence of graft rejection based on biopsy reports revealed that most (72%) showed ACR [19]. The impact of CMV infections on graft functions in post-renal transplant recipients revealed that 31 (67%) were associated with chronic graft dysfunction, 11 (24%) were associated with acute graft dysfunction, and four (9%) showed stable graft function, which is due to the inability to adequately treat acute rejection due to the presence of CMV disease or the increased virulence of latent CMV virus in recipients being treated for acute rejection. So suggested a role for more aggressive prophylaxis against CMV disease, especially at the time of treatment for acute rejection [20]. This differs from another study, which states that transplant patients with CMV disease had a significant likelihood of developing acute rejection after CMV infection or reactivation (P<0.01) [21].

In this study, the comparison of graft dysfunction in primary infection/reactivation is assessed, which shows that in primary infection, acute graft dysfunction is more common. In the case of reactivation, chronic graft dysfunction is more common. Immunologic monitoring plays a promising role in identifying patients with the potential of progressing unfavorably and it is a tool that should be added to clinical practice on a large scale [22].

Limitations of the study

Considering the prevalence of CMV infection in our setting, the duration of study the sample size was 46. A larger sample size will give more data aiding in better analysis.

Strengths

Integrating IgG avidity testing into routine screening aids in identifying potential recipients at risk for developing CMV infection, thus helping to prevent potential graft dysfunction.

## Conclusions

To conclude, this study emphasizes the profound impact of CMV on graft function in post-renal transplant recipients. With a focus on the microbiological dimensions and the critical role of CMV antibody screening, it underscores the necessity of vigilant monitoring and prophylactic antiviral therapy to reduce CMV infection risks and enhance patient outcomes. The study's findings highlight the essential function of IgG avidity testing in differentiating between primary infection and reactivation, facilitating timely and effective interventions to prevent graft dysfunction and rejection.
